# Effectiveness-implementation of COPD case finding and self-management action plans in low- and middle-income countries: global excellence in COPD outcomes (GECo) study protocol

**DOI:** 10.1186/s13063-018-2909-8

**Published:** 2018-10-19

**Authors:** Trishul Siddharthan, Suzanne L Pollard, Shumonta A Quaderi, Andrew J Mirelman, Maria Kathia Cárdenas, Bruce Kirenga, Natalie A Rykiel, J Jaime Miranda, Laxman Shrestha, Ram K Chandyo, Adithya Cattamanchi, Susan Michie, Julie Barber, William Checkley, John R Hurst, GECo Study Investigators, Shumonta Quaderi, Susan Michie, John R Hurst, Zachos Anastasiou, Julie Barber, Trishul Siddharthan, Suzanne L Pollard, Brooks Morgan, Natalie A Rykiel, Mathew Grigsby, Nicole Robertson, Robert A Wise, William Checkley, Laxman Shrestha, Karbir Nath Yogi, Arun Sharma, Ram K Chandyo, Patricia Alupo, Denis Muwonge, Denis Mawanda, Faith Nassali, Robert Kalyesubula, Bruce Kirenga, Andrew J Mirelman, Marta Soares, Oscar Flores-Flores, Elisa Romani-Huacani, Maria Kathia Cárdenas, J Jaime Miranda, Adithya Cattamanchi

**Affiliations:** 1 Division of Pulmonary and Critical Care, School of Medicine, Johns Hopkins University, Baltimore, OH USA; 2 Center for Global Non-Communicable Diseases, School of Medicine, Johns Hopkins University, Baltimore, OH USA; 3 UCL Respiratory, University College London, London, UK; 4 Centre for Health Economics, University of York, York, UK; 5 CRONICAS Centre of Excellence in Chronic Diseases, Universidad Peruana Cayetano Heredia, Lima, Peru; 6 Makerere Lung Institute, Makerere University, Kampala, Uganda; 7 Departamento de Medicina, Facultad de Medicina, Universidad Peruana Cayetano Heredia, Lima, Peru; 8 Institute of Medicine, Tribhuvan University, Kathmandu, Nepal; 9 Kathmandu Medical College, Kathmandu, Nepal; 10 Division of Pulmonary and Critical Care Medicine, University of California, San Francisco, CA USA; 11 Centre for Behaviour Change, University College London, London, UK; 12 Department of Statistical Science, University College London, London, UK

**Keywords:** COPD, COPD exacerbations, COPD case finding, COPD action plan, Non-communicable disease, Self-management

## Abstract

**Background:**

Chronic obstructive pulmonary disease (COPD) is the end result of a susceptible individual being exposed to sufficiently deleterious environmental stimuli. More than 90% of COPD-related deaths occur in low- and middle-income countries (LMICs). LMICs face unique challenges in managing COPD; for example, deficient primary care systems present challenges for proper diagnosis and management. Formal diagnosis of COPD requires quality-assured spirometry, which is often limited to urban health centres. Similarly, standard treatment options for COPD remain limited where few providers are trained to manage COPD. The Global Excellence in COPD Outcomes (GECo) studies aim to assess the performance of a COPD case-finding questionnaire with and without peak expiratory flow (PEF) to diagnose COPD, and inform the effectiveness and implementation of COPD self-management Action Plans in LMIC settings. The ultimate goal is to develop simple, low-cost models of care that can be implemented in LMICs. This study will be carried out in Nepal, Peru and Uganda, three distinct LMIC settings.

**Methods/design:**

We aim to assess the diagnostic accuracy of a simple questionnaire with and without PEF to case-find COPD (GECo1), and examine the effectiveness, cost-effectiveness and implementation of a community-health-worker-supported self-management Action Plan strategy for managing exacerbations of COPD (GECo2). To achieve the first aim, we will enrol a randomly selected sample of up to 10,500 adults aged ≥ 40 years across our three sites, with the goal to enrol 240 participants with moderate-to-severe COPD in to GECo2. We will apply two case-finding questionnaires (Lung Function Questionnaire and CAPTURE) with and without PEF and compare performance against spirometry. We will report ROC areas, sensitivity and specificity. Individuals who are identified as having COPD grades B–D will be invited to enrol in an effectiveness-implementation hybrid randomised trial of a multi-faceted COPD self-management Action Plan intervention delivered by CHWs. The intervention group will receive (1) COPD education, (2) facilitated-self management Action Plans for COPD exacerbations and (3) monthly visits by community health workers. The control group will receive COPD education and standard of care treatment provided by local health providers. Beginning at baseline, we will measure quality of life with the EuroQol-5D (EQ-5D) and St. George’s Respiratory Questionnaire (SGRQ) every 3 months over a period of 1 year. The primary endpoint is SGRQ at 12 months. Quality-adjusted life years (QALYs) using the Short-Form 36 version 2 will also be calculated. We will additionally assess the acceptability and feasibility of implementing COPD Action Plans in each setting among providers and individuals with COPD.

**Discussion:**

This study should provide evidence to inform the use of pragmatic models of COPD diagnosis and management in LMIC settings.

**Trial registration:**

NCT03359915 (GECo1). Registered on 2 December 2017 and NCT03365713 (GECo2). Registered on 7 December 2017. Trial acronym: Global Excellence in COPD Outcomes (GECo1; GECo2).

**Electronic supplementary material:**

The online version of this article (10.1186/s13063-018-2909-8) contains supplementary material, which is available to authorized users.

## Background

### The global importance of COPD

Chronic obstructive pulmonary disease (COPD) is ‘*a common, preventable and treatable disease that is characterized by persistent respiratory symptoms and airflow limitation that is due to airway and/or alveolar abnormalities usually caused by significant exposure to noxious particles or gases*’ [1]. The primary risk factor for COPD in high-income countries (HICs) is tobacco smoke exposure; however, household air pollution (HAP), from burning solid fuels such as wood, dung, agricultural crop waste, and coal for energy, is the primary risk factor for COPD in low- and middle-income countries (LMICs) [2]. In addition to chronic progressive symptoms and functional impairment, some individuals with COPD are prone to intermittent deteriorations in respiratory health, or ‘exacerbations’, often driven by infection [3]. The global burden of COPD is large and increasing. In 2015, it was estimated that 174 million people worldwide had clinically significant COPD, and an estimated 3.2 million individuals died from the disease, an increase of 11.6% since 1990 [4]. COPD will become the third leading cause of death by 2030 [5]. Prevalence estimates vary due to different methods of diagnosis; however, the BOLD studies report a prevalence of moderate COPD or higher to be around 10% globally [1, 6]. Exacerbations are a major source of morbidity from COPD, and the cause of direct healthcare costs in high-income countries [7].

### COPD in LMICs

More than 90% of COPD-related deaths occur in LMICs [8]. The economic cost of illness due to COPD among LMICs was estimated to be US$1 trillion in 2010 and is expected to increase to US$2.6 trillion by 2030 [9]. Indirect costs, including loss of productivity both by individuals affected by COPD and their caregivers, are important in LMICs [9]. LMICs face unique challenges when addressing COPD, including poorly resourced primary care systems and trained workforce shortages, which present challenges with COPD diagnosis and management, especially during exacerbations [10]. The chronic nature of COPD means that people may access multiple healthcare providers, including alternative providers.

### COPD case finding

The ‘gold standard’ method for diagnosis of COPD is quality-assured, post-bronchodilator spirometry, though COPD represents a range of phenotypes with different symptomatic presentations including shortness of breath, cough and sputum production [1]. In LMICs this is often only available from pulmonary physicians in specialised urban centres, while most of the burden associated with this condition occurs in rural areas [10]. A number of COPD case-finding questionnaires have been validated in HIC settings, which are, therefore, likely to be more sensitive to tobacco-associated COPD than biomass-fuel smoke [11–13]. In 2010, Yawn et al. developed and validated a simple five-item Lung Function Questionnaire (LFQ) and compared this to standard spirometry (area under the curve (AUC) = 0.720 with sensitivity and specificity of 73.2 and 58.2%, respectively) [11]. Martinez et al. combined peak expiratory flow (PEF) measurements with a case-finding instrument to improve the sensitivity, specificity and AUC for detecting COPD (89.7%, 78.1%, 0.795, respectively) [12]. Case-finding instruments combined with low-cost peak-flow meters could be useful tools for identifying individuals who should be further screened for COPD in LMIC settings as well, although this has not been previously tested.

### COPD self-management

Written self-management Action Plans are an integral part of evidence-based management of COPD according to international guidelines [1]. Action Plans support people with COPD to recognise and react appropriately to exacerbations through initiation of additional pharmacotherapy and modification of healthcare-seeking behaviours [14]. A number of studies and systematic reviews have tested self-management interventions for prevention and successful treatment of COPD exacerbations in high-income settings [15–19]. Bourbeau et al. demonstrated a 39.8% reduction in hospitalisations for exacerbations of COPD in a multicentre randomised control trial of self-management with comprehensive patient education compared to usual care [14]. A similar trial by Effing et al. reviewed the effectiveness of self-management on the severity of exacerbations and found a trend to fewer exacerbation days [20]. Finally, a 2005 Cochrane Review found that Action Plans aid people with COPD in recognising severe exacerbations and reacting appropriately to an exacerbation via the self-initiation of antibiotics and/or orally administered steroids [16]. The potential benefits, cost-effectiveness and implementation challenges of Action Plans in LMIC have not previously been studied.

Efforts to scale-up COPD case finding and management are hampered by a shortage of specialised healthcare providers. Healthcare workforce shortages have been well documented among LMICs and community health worker (CHW) models of care have been widely utilised in health priority areas including infectious diseases and nutritional interventions [10, 21]. CHWs are a diverse category of health workers who commonly work in communities outside of fixed health facilities and have formal, but limited, training for the tasks they perform [21]. CHWs can serve as a bridge between people and healthcare systems [22]. Many LMICs have existing CHW networks and a long history of implementing public health interventions utilising CHWs to deliver health services [21]. CHWs are uniquely qualified as connectors and tools of empowerment in chronic disease management because they live within the community in which they work [22–24].

Given the high and rising global burden of COPD, better strategies to diagnosis COPD and manage exacerbations are urgently needed for LMICs. In two linked studies (Global Excellence in COPD Outcomes: GECo1 and GECo2), we aim to validate a modified COPD case finding questionnaire (with and without PEF) to better identify individuals for further screening for COPD, as well as to develop evidence to support the wider implementation of COPD Action Plans among CHWs, allowing for simple, low-cost models of COPD care in LMICs. This study will enrol individuals in three distinct LMIC regions, namely Nepal, Peru and Uganda.

## Method / design

### Goals

**Fig. 1:**
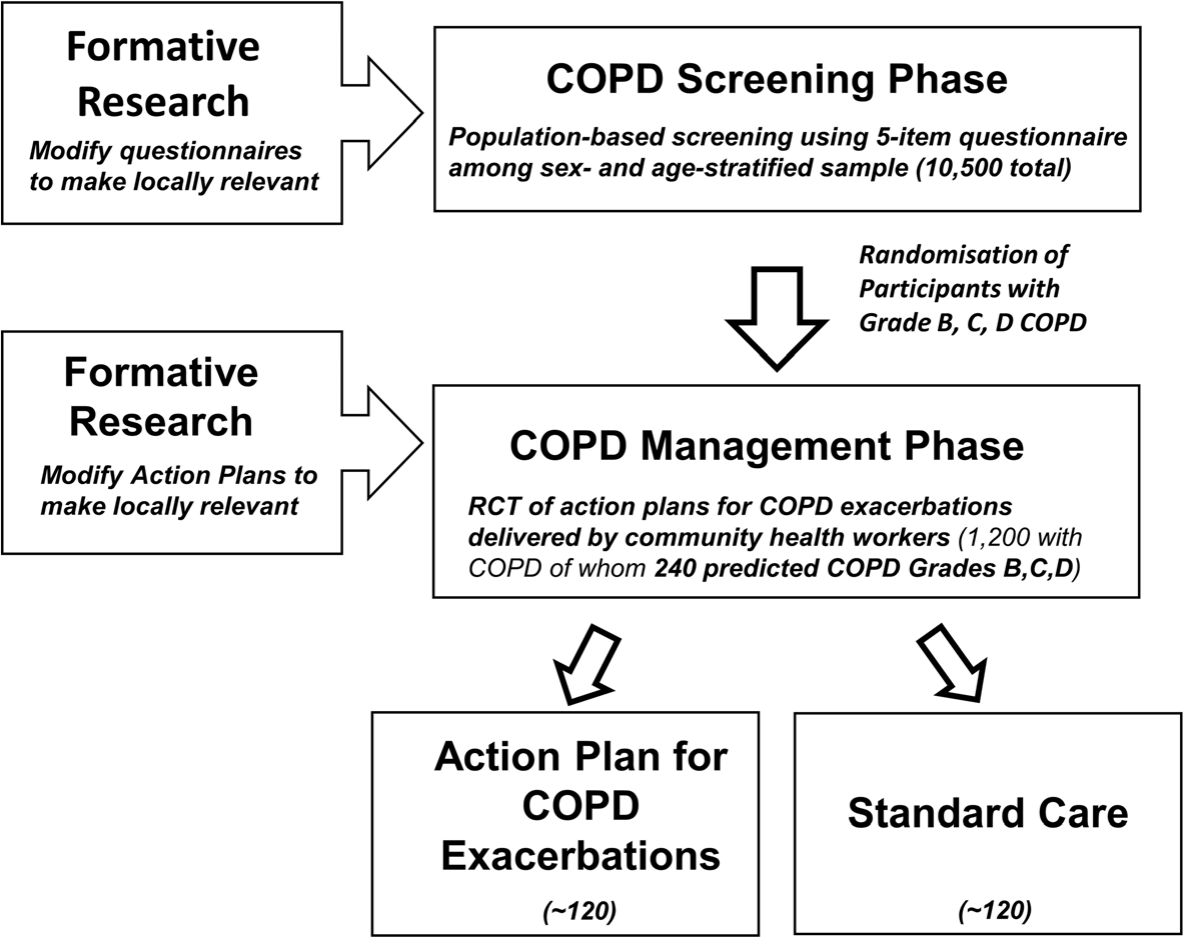
Overview of the GECo Studies

**Fig. 2:**
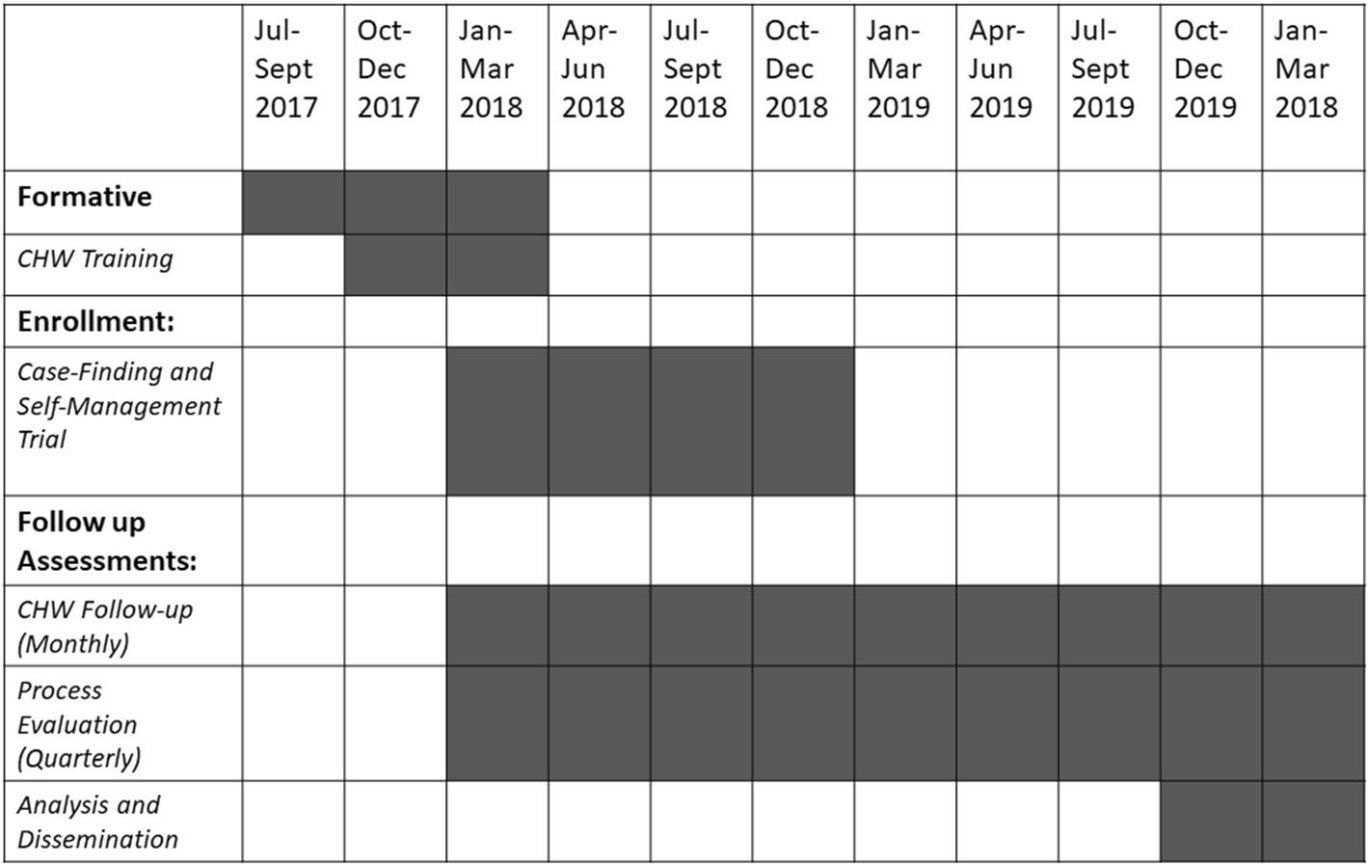
Schedule of enrolment, interventions and assessments

The over-arching goal of the GECo studies is to develop simple, cost-effective models of COPD care that can be implemented in LMICs. An overview of the studies is shown in Fig. 1. Their schedule of enrolment, interventions and assessments in shown in Fig. 2.

### Objectives

The objectives of the present study are to validate case-finding instruments with and without peak-flow measurements in three diverse LMIC settings, and to develop evidence supporting the effectiveness, cost-effectiveness and implementation of a CHW-based strategy to deliver self-management Action Plans for COPD.

### Research questions

#### Primary research questions

1. GECo1: What is the diagnostic accuracy of case finding for COPD using a questionnaire with and without peak-flow measurements, compared to gold-standard spirometry?
2. GECo2: What is the effect of a multi-faceted intervention comprising a self-directed COPD Action Plan delivered and supported by CHWs for the management of COPD exacerbations on respiratory health, and is this intervention likely to be implementable and cost-effective?

#### Implementation research questions

1. GECo1: What is the appropriateness, acceptability and feasibility of using questionnaires to identify COPD cases from the perspective of local community members, community health workers, local health centre physicians and Ministries of Health?
2. GECo2: What is reach, adoption, implementation and maintenance of a self-directed COPD Action Plan strategy for management of COPD exacerbations?
3. GECo2: How does the value for money of the self-directed COPD Action Plan strategy vary by different implementation constraints and for different socioeconomic sub-groups?

### Study design overview

#### GECo1

For the *Case-finding study* (GECo1) we will test the diagnostic accuracy of case-finding instruments in LMIC settings. To achieve the aim we will enrol a representative community sample of up to 10,500 adults of 40 years of age and above in Nepal, Peru and Uganda (Fig. 2). We will apply two modified questionnaires, with and without PEF measurements, and compare performance of this testing to spirometry, which will be conducted in the field according to the American Thoracic Society/European Respiratory Society (ATS/ERS) standards using Easy-on-Air spirometers (ndd, Zurich, Switzerland). The primary endpoint is area under the receiver operating characteristics (ROC) curve. We will report the sensitivity, specificity and ROC of case-finding instrument with and without PEF compared to standard spirometry.

#### GECo2

For the *Self-management trial* (GECo2), we will randomise 240 adults (total for all sites) with grades B–D COPD as per the GOLD 2017 classification, identified in the case-finding phase, to an intervention or control arm. We will develop and test a locally adapted, CHW-based intervention strategy to improve self-management of COPD (see ‘Description of intervention’). CHWs will be instructed on chronic disease management for COPD, and how to utilise the self-management Action Plan for management of exacerbations. We will measure quality of life with the EuroQol-5D (EQ-5D) and St. George’s Respiratory Questionnaire (SGRQ) every 3 months over a period of 1 year. The primary efficacy endpoint is the change in the SGRQ at 12 months. Results of the EQ-5D will be used to calculate quality-adjusted life years (QALYs) which will inform the analysis of the cost-effectiveness and equity impacts of implementation. We will additionally assess the reach, adoption, implementation and maintenance of Action Plans for exacerbations in each setting.

### Settings

The study settings represent three distinct geographic and economic regions in Asia, Latin America and Sub-Saharan Africa. Inclusion of these countries will allow us to assess varying degrees of urbanisation, environmental exposures (i.e. tobacco and biomass-fuel smoke), and varying implementation contexts.

*Nepal* is a low-income country located in Southeast Asia with a total population of 26.5 million, of which 82% is rural. Nepal’s GDP is £49 billion, with 25% of the population living below the national poverty line. The current ratio of physicians per person is 1:1742. The current minimum wage is Rs 9700 per month (£66). The study site is in Bhaktapur, 8 miles east of Kathmandu. The majority of the estimated 80,000 inhabitants of Bhaktapur municipality are either craftsman or businessmen, while many migrants come to work in the outskirts at brick or carpet factories. CHWs, called female community health volunteers (FCHVs), are volunteers coordinated by the Bhaktapur municipality to promote community-based healthcare, health education and referrals. FCHVs are trained by their District Public Health Office, under the Ministry of Health to serve within their respective communities, mainly on family planning, vaccination and nutrition programmes. There are approximately 90 FCHVs active in the municipality of Bhaktapur and over 52,000 throughout Nepal.

*Peru* is an upper-middle-income country located in South America with a population of 30.5 million, 10 million of whom live in the capital (Lima), and 78.6% of whom live in urban areas. Peru’s GDP is £145 billion, and 26% of the population lives below the national poverty line. The current ratio of physicians per person is 1:1116. The minimum wage in Peru is 850 soles per month (£186). We will conduct this study in Pampas de San Juan de Miraflores, a peri-urban community in southern Lima, Peru’s capital. CHWs or ‘*agentes comunitarios*’ include community members who volunteer to support health education programmes and campaigns at their corresponding health centre. The size of CHW networks and specific duties and responsibilities can vary considerably depending on the region, district, or health centre.

*Uganda* is a low-income country located in East Africa with a total population of 37 million, and a large rural population (> 80%). Uganda’s GDP is £19 billion with 19.5% living below the national poverty line. The study will be carried out in the Nakeseke District of Uganda. Most of the inhabitants (75%) are subsistence farmers and over 60% of them live on less than 45,000 shillings (£9) per month. The current ratios of physicians and nurses per person are 1:25,000 and 1:5000, respectively, making Nakeseke one of the most under-resourced health districts in Uganda. CHWs called Village Health Teams (VHTs) are selected in each village by the Uganda Ministry of Health. They provide formal referral services to local health centres and assist with community-based follow-up. The VHT consist of community members who volunteer for the position and are trained by the Uganda Ministry of Health.

### Study populations

We will enrol an age- and sex-stratified random sample of full-time residents of the proposed study areas in Nepal, Peru and Uganda using existing census data. Inclusion criteria are: aged ≥ 40 years; capable of performing spirometry; and being a full-time resident of the community. Full-time residence will be defined as having lived in the study area for more than 6 months. Exclusion criteria are: self-reported pregnancy; having active pulmonary tuberculosis or being on medications for pulmonary tuberculosis; unable to do spirometry because of eye surgery, thoracic surgery, abdominal surgery, or myocardial infarction in the 3 months prior to study visit or a blood pressure > 180/100 mmHg. For the self-management component of the trial, we will enrol individuals who were identified to have grades B–D COPD [1].

### Procedures case-finding phase (GeCo1)

#### Data collection

Demographic questionnaires will be applied to obtain socioeconomic information, exposure history to cigarettes and household air pollution, medical history and family history of respiratory illness. Data will be collected by trained field workers at each site and will be electronically entered into REDCap using tablets with GPS capability (Asus Z380M ZenPad, Taipei, Tawain) [25].

##### Lung Function Questionnaire (LFQ)

We will administer an instrument context-adapted from the original LFQ, which has been validated in high-income countries, and apply it to LMIC settings [11]. The LFQ assesses five items: age, smoking history, wheeze, dyspnoea and phlegm. The modified questionnaire will include additional items including exposure to biomass fuel and will be administered by field workers (see Additional file 1).

##### Capture

CAPTURE is a simple five-item questionnaire which, together with PEF, has been shown to be a viable approach for COPD case identification in the US in primary care settings [12]. CAPTURE with PEF can identify COPD patients who would benefit from currently available therapy and require further diagnostic evaluation, and we will use this validated instrument and apply it to LMIC. CAPTURE assesses five items: environmental exposure, sensitivity to air quality/weather, how breathing interferes with physical activities, comparing health with peers, and exacerbations.

##### *MRC Dyspnoea Scale and COPD Assessment Test* (CAT)

At the case-finding visit, participants will be asked to complete the modified Medical Research Council Dyspnoea Score (mMRC) and the COPD Assessment Test (CAT), which have been translated into relevant local languages and previously validated. The mMRC categorises self-perceived disability among those with COPD on a five-item scale. The CAT is designed to measure the impact of COPD on a person’s well-being and daily life and is measured with eight items on a 40-point scale and will be administered to those with COPD on spirometry.

##### Anthropometry and spirometry

All participants will have blood pressure, weight and standing height and spirometry performed**.** Anthropometric measurements will be recorded in triplicate and the median measurements will be used for analysis. Systolic and diastolic blood pressure measurements on the second and third measurements will be averaged to calculate blood pressure; the first measurement will be ignored to avoid potential white-coat hypertension.

Trained study fieldworkers will conduct spirometry using a flow-based portable spirometer to measure pulmonary function and will record forced vital capacity (FVC), forced expiratory volume in 1 s (FEV_1_), the percentage of FVC exhaled in the first second (FEV_1_/FVC), and flow-volume curves. We will obtain at least three acceptable manoeuvres in accordance with ATS/ERS guidelines [26]. We will use the Global Lung Function Initiative mixed ethnic population reference for calculation of percentage predicted values or Z-scores [27]. We will test for reversibility (increase in FEV_1_ of ≥ 12% and increase in FEV_1_ ≥ 200 mL) with two puffs from a salbutamol inhaler (90 mcg/puff) via a spacer. A COPD diagnosis will be defined as post-bronchodilator FEV_1_/FVC below the lower limit of normal for that population following ATS/ERS standardised guidelines [26].

##### Spirometry quality control

All spirometry will be read by two independent reviewers locally who have been trained in spirometry per ATS/ERS guidelines [27]. Spirometry that is deemed not acceptable or reproducible will be repeated up to one additional time. If there is discrepancy in local reviewers over reads, the spirogram in question will be reviewed centrally. Additionally, 10% or all curves will be independently reviewed centrally for site-specific quality control. Spirometry will be graded according to ATS/ERS classification and only high- quality spirometry will be included for analysis and trial recruitment [28].

#### Sample size and data analysis

##### Sample size

The sample size required to estimate the ROC area within 1.5% (based on a 95% confidence interval), assuming an anticipated sensitivity of 90% and specificity of 60%, and assuming 11% of those screened will have COPD is 9669 participants [2, 29]. To ensure an adequate sample size is subsequently available for GECo2, we will recruit a total of up to 10,500 subjects (3500 at each site).

##### Analysis

By site and overall we will summarise the characteristics of those consenting to the study including demographics, exposure history to tobacco and/or household air pollution, anthropometric measurements, spirometry measurements (FEV_1_, FVC and FEV_1_/FVC ratio) and lung function scores (mLFQ, Capture, mMRC dyspnoea scores).

Using a receiver operating characteristic (ROC) area analysis, we will examine the diagnostic accuracy of the questionnaire scores in identifying cases of COPD (compared to spirometry). Curves will be obtained for the mLFQ and CAPTURE questionnaires alone and then each with addition of the PEF scores. Logistic regression models will be used to obtain the ROC curve and area (AUC), with 95% confidence interval. Estimates will be weighted based on census information from each site to better reflect the population. A comparison between the ROC areas will be made by site.

#### Self-management trial (GeCo2)

We invite participants with grades B–D COPD from the case-finding phase to participate in the randomised controlled trial in which they will be randomised to either a multi-faceted intervention to promote adoption of self-management of COPD exacerbations or continue to follow usual care practices for COPD management. Full study information will be provided and all patients will provide written informed consent.

#### Randomisation

Once a participant is determined to be eligible and agrees to enter the study, they will be randomised using an online system (https://www.sealedenvelope.com/) at each local site by the site-specific research team. The computerised randomisation system will use 1:1 allocation to the enhanced support and usual care groups, stratified by country and using random permuted blocks of variable sizes. The assignment allocated to each participant will be recorded in REDCap at local sites by the local research team.

#### Blinding

Principal investigators and members of the data coordinating centre will be blinded to treatment allocation.

#### Intervention arm

The intervention arm will receive (1) basic COPD education, (2) CHW-delivered training in self-management using a locally adapted Action Plan, (3) free CHW-delivered rescue pack medications (steroid, antibiotic) for acute exacerbations (as compared to free rescue medications from local health facilities in the control group). In addition, the intervention arm will receive (4) monthly home visits and (5) continuous access to a CHW with expertise in educating, advising and reinforcing self-management behaviours. (Table 1).

**Table 1:** Multi-component strategy for self-directed management of chronic obstructive pulmonary disease (COPD) exacerbations

Component	Description
COPD education (control and intervention arms)	Basic COPD-specific education on (1) risk factors (e.g. household air pollution and tobacco), (2) symptoms and disease progression and (3) how to avoid exacerbations and maintain general lung health (exposure reduction, exercise, sleep and nutrition) using modified versions of standardised educational tools (e.g. ‘flip charts’) administered by trained CHWs
Facilitated self-management Action Plan	2-page Action Plans which colour-code states of respiratory health into green, yellow and red zones. (1) The green zone describes the patient’s baseline respiratory symptoms and actions emphasise daily healthy behaviour and harm reduction strategies, (2) The yellow zone denotes worsening dyspnoea and signifies an exacerbation warranting use of inhaled bronchodilators and orally administered corticosteroids. If there is a change in sputum quantity or colour the addition of antibiotics to corticosteroids is suggested, (3) The red zone pertains to profound dyspnoea or associated symptoms including chest pain, fevers, haemoptysis or change in mentation and urgent medical advice is recommended. Action plans will be facilitated by contact with assigned community health workers
Monthly visitation by CHWs	CHWs will reinforce concepts from the initial COPD education and the Action Plan during monthly visits. CHWs will assess the individual’s ability to recognise symptoms and take appropriate action as indicated by the Action Plan and will be trained to provide feedback and reinforcement accordingly. CHWs will provide additional rescue packs to participants as needed

*CHW* community health worker

#### Control arm

The control arm will receive (1) basic COPD education (see ‘Additional file 1’), (2) access to free medications (steroids, antibiotics) for acute exacerbations available at a local health facility and (3) access to usual care in the local setting. Usual care is as follows per local practice:

##### Nepal

Participants with grades B–D COPD will be referred to the local health centres in their respective catchment areas. They will have access to their local CHW per usual care, though this CHW would not have had extra training or materials pertaining to self-management Action Plans. If indicated by their healthcare provider, medications will be provided by the study team for COPD exacerbation from the Khwopa Public Health Care Centre, our study site.

##### Peru

Participants identified with grades B–D COPD from the case-finding phase (GECo1) will be referred to local health centres in their respective catchment areas. If indicated by their healthcare provider, medications will be provided by the study team for COPD exacerbations.

##### Uganda

Participants with grades B–D COPD will have access to their local CHW per usual care, though this CHW would not have had extra training or materials pertaining to self-management Action Plans. Participants will be referred to local health centres in their respective catchment areas. If indicated by their healthcare provider, medications will be provided by the study team for COPD exacerbation.

#### Formative phase

The intervention strategy was informed by the results of formative research efforts conducted between July 2016 and October 2017. Formative research efforts included in-depth interviews carried out among community participants with COPD, CHWs and healthcare providers/Ministry of Health officials across the three field sites. As part of these efforts, we explored local terminology, perceptions and explanatory models among community members and healthcare practitioners, characterised the supervisory and incentive structures of CHW networks to determine feasibility of a CHW-delivered intervention strategy in each setting, identified, categorised and prioritised multi-level barriers and facilitators to target using the COM-B/Behaviour Change Wheel framework [30]. We then selected intervention functions and behaviour change techniques that targeted the key barriers to adoption to arrive at our final intervention strategy.

#### Intervention design

This CHW-based intervention strategy centred on delivery of locally adapted self-management Action Plans was designed to promote adoption of basic self-management practices and health-seeking behaviours during an acute exacerbation among individuals with COPD. Specifically, the intervention is targeted toward modification of the following health behaviours: (1) monitoring and recognition of warnings signs of a COPD exacerbation and (2) taking prescribed rescue medications and/or seeks attention at a health facility as indicated by evidence-based guidelines.

#### Intervention components

##### CHW-delivered COPD education

All individuals enrolled in the self-management trial (GECo2) (control and intervention) will receive basic COPD education delivered by local CHWs. CHWs will provide COPD-specific education on risk factors (e.g. household air pollution and tobacco), symptoms and disease progression. Additionally, they will be advised on how to avoid exacerbations and maintain general lung health (exposure reduction, exercise, sleep and nutrition). We will use modified versions of standardised educational tools (e.g. ‘flip charts’), which have been previously developed and evaluated in Uganda [31]. Over the course of the trial we will assess the delivery of the health intervention through quarterly fidelity assessments which will take place during monthly CHW visits.

##### Self-management Action Plan

**Fig. 3:**
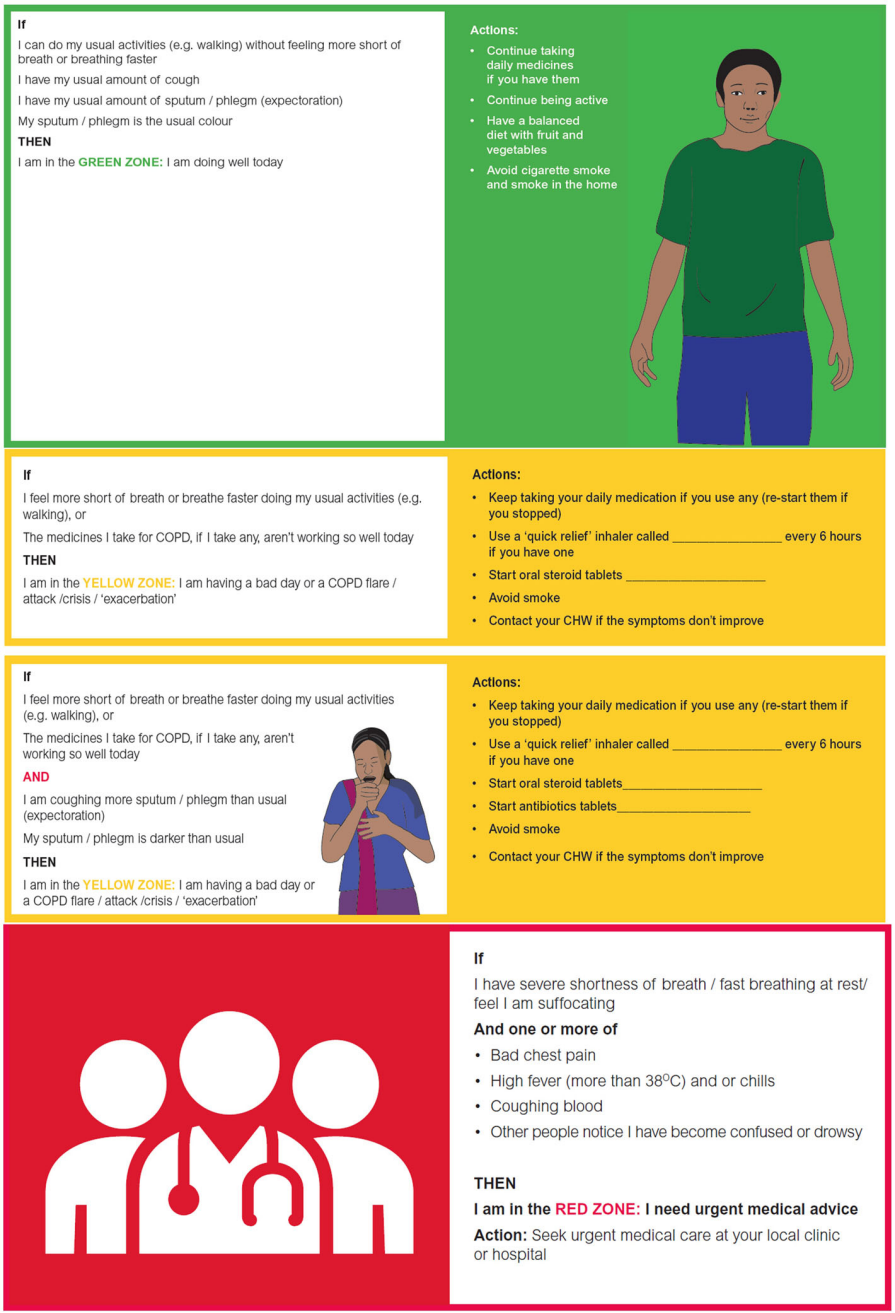
Chronic obstructive pulmonary disease (COPD) self-management Action Plan. Action plans will be distributed to the intervention arm of trial in addition to medication rescue packs. Sections are colour-coded (green – usual care, yellow – COPD exacerbation self-management, red – urgent medical care)

We developed the COPD Action Plan based on existing Action Plans from the UK National Health Service and the American Lung Association for use in each of the three country sites [32]. The Action Plan is identical across the sites, apart from use of local language and illustrations. The Action Plan includes instructions for symptom awareness and medication self-management, with recommendations for tobacco cessation and exposure reduction, pulmonary rehabilitation, exercise, nutrition and sleep, and local health centre contact information. Adaptation of the educational tools (e.g. flip charts) and the COPD Action Plan were informed by the results of formative research efforts. We incorporated local terminology and tailored visual representations of COPD risk factors, pathophysiology, and symptoms to local context (Fig. 3). After the creation of an initial prototype, we then solicited feedback about Action Plan content and layout through consultation with local healthcare practitioners and community members across the sites and updated it accordingly.

##### Follow-up visits and reinforcement of self-management practices by CHWs

Follow-up visits by CHWs will occur monthly for the intervention arm. During these visits, CHWs will reinforce concepts from the initial COPD education and the Action Plan. CHWs will assess the individual’s ability to recognise symptoms and take appropriate action as indicated by the Action Plan, and will be trained to provide feedback and reinforcement accordingly. Finally, CHWs will assess medication use through self-report instruments and pill counts, and provide additional rescue packs as needed. CHWs will keep detailed records of contacts with participants.

##### Access to CHW-delivered rescue packs for acute exacerbations

**Fig. 4:**
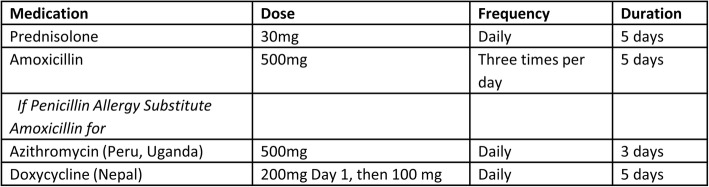
Rescue pack for chronic obstructive pulmonary disease (COPD) exacerbations. Medications based on GOLD recommendation for treatment of COPD exacerbations and local prescribing practices

Both the intervention and control arms will receive access to rescue medications for COPD. CHWs will deliver rescue packs (prednisone and antibiotics – local choice) to intervention arm participants at the beginning of the trial and as needed (Fig. 4). Control arm participants will receive either free rescue packs from a local pharmacy or be reimbursed for rescue medications purchased during the trial, depending on local norms and requirements. The efficacy of corticosteroids and antibiotics for COPD exacerbations has been previously well documented. We intend to specifically assess the effectiveness of a multi-faceted intervention to promote self-management of COPD exacerbations at a community level. While both study arms will receive medications for management of COPD exacerbations, we hypothesise that a multi-faceted strategy of self-directed care will lead to improved health outcomes and be cost-effective.

#### Role of the CHWs

CHWs will deliver basic COPD education to all individuals who are diagnosed with COPD in the case-finding phase (GECo1). CHWs will additionally support the self-management action plan, of those participants recruited to the self-management trial (GECo2) and randomised to the intervention arm via monthly visits and distribution of rescue packs for COPD when required. The CHWs will have three roles:

- *Role 1*: relevant during GECo1 and throughout the study
  - The CHW will deliver COPD education within 72 h of COPD diagnosis via educational flip-charts. There will be no mention of self-management Action Plans, just basic COPD education delivery via flip charts (see ‘Additional file 1’)
- *Role 2*: relevant during GECo2
  - The CHWs will be trained by the research team to support the self-management Action Plans. They will be taught how to teach participants to use the Action Plan; symptoms to be mindful of; and to remind participants to partake of daily physical activity, eat well and sleep well. They will also issue participants in the intervention arm a rescue pack of antibiotics and steroids, educate them when and how to take them and reinforce that they are contactable daily for advice regarding COPD and self-management
- *Role 3*: relevant during GECo2
  - The CHWs will conduct monthly visits to the participants’ homes in the intervention arm (approximately one participant per week per CHW). At each visit they will reiterate the education listed above and re-emphasise the role and use of the Action Plan. They will re-stock medications if a rescue pack has been used. They will record if a pack has been used, when it was used, and whether the medications were initiated correctly and the course was completed. They will record what symptoms the participant experienced that prompted them to take the medications in each instance. Monthly visits by the CHWs to the intervention arm participants to support the Action Plan will occur to aid in promotion of behavioural changes

#### CHW training and health education

CHWs will be selected from local catchment areas and trained on delivering health education related to COPD based on willingness to participate. CHWs will participate in a 2-day workshop consisting of health education and role-playing which is harmonised across sites (see ‘Additional file 1’). CHWs assigned to the intervention component of the trial will additionally receive training on how to support individuals with COPD in self-management of symptoms and exacerbations as indicated in the Action Plans. CHWs delivering the intervention arm will also be trained in medication distribution, patient communication, navigation of local health systems, and on delivering reinforcement of self-management behaviours during follow-up (Training plan included in the ‘Additional file 1’). A COPD Knowledge Questionnaire will be applied before and after training to assess the effectiveness of the delivered training to the CHW. CHWs will be evaluated for a set of specific competencies before being approved to interact with study participants. Training will be repeated twice through the study to reinforce concepts taught in initial training and address questions or concerns that arise during the follow-up period.

### Follow-up data collection

**Fig. 5:**
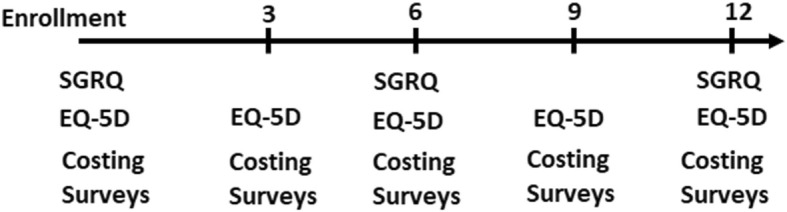
GECo 2 follow-up. Participants will be followed quarterly and queried on exacerbation history and healthcare utilisation. St. George’s Respiratory Questionnaire (SGRQ) will be administered 6-monthly, EuroQol-5D and health-costing surveys will be administered quarterly

Data will collected by field workers at baseline and at the 3-, 6-, 9- and 12-month follow-ups (from baseline) (Fig. 5). The baseline data is as collected at the GECo1 visit. It is important to note that the 3-monthly visits from the research staff are not the same as the monthly CHW visits in the intervention arm.

#### *St. George’s Respiratory Questionnaire (SGRQ)* (primary outcome)

The primary outcome of the trial will be a comparison of the change in SGRQ from baseline to 12 months between the two arms [33]. We will assess respiratory symptoms at baseline, 3, 6, 9 and 12 months post randomisation using the SGRQ. The SGRQ measures impaired health and perceived well-being among individuals with chronic airway disease and offers many advantages for our study, namely: (1) can be used to quantify changes in health following treatment, (2) it is not limited to individuals with COPD and (3) it provides a standard metric that can be used for easy comparison across our three diverse settings [33, 34].

The EuroQol-5D (EQ-5D) questionnaire will be collected at baseline and quarterly through the trial period. The EQ-5D is a generic instrument for describing and valuing health. It is based on a descriptive system that defines health in five dimensions: Mobility, Self-Care, Usual Activities, Pain/Discomfort and Anxiety/Depression [35]. Each dimension has three response categories corresponding to no problems, some problems and extreme problems. The instrument is designed for self-completion, and respondents also rate their overall health on the day of the interview from a 0–100 hash-marked, visual analogue scale (EQ-VAS). Quality-adjusted life years (QALYs) will be derived from the EQ-5D. The EQ-5D has been widely tested and used in both general populations and patient samples and has been locally validated in Nepal, Peru and Uganda [35].

##### Health economics questionnaire

We will collect detailed information on health-related outcomes including number of exacerbations, hospitalisations as well as healthcare costs and health-related productivity costs at 3-monthly follow-up visits.

##### Medication use

We will record pill counts of antibiotics and steroids at quarterly follow-up visits and ask about self-reported frequency and duration of respiratory medications.

#### Sample size and data analysis

##### Sample size

We calculated the sample size required for this trial using a confidence interval approach justified and described elsewhere [36]. This approach aims to ensure an adequate sample size to help inform a decision about continuation of GECo2 to a main trial based on an indication of a treatment effect for the primary outcome. This SGRQ score has previously been shown to have a standard deviation of 25 points in a similar population and the clinically important difference is 4 points (a recent Cochrane Review on the use of COPD Action Plans found an average decrease of 4 points in SGRQ in three randomised trials) [16]. A sample of 112 participants with COPD will be needed to produce an 80% one-sided confidence interval that excludes a 4-point difference in SGRQ under the scenario of no difference in means. We expect to have sufficient subjects to enrol 240 participants from the GECo1 study.

##### Analysis

The primary analysis will be a comparison of SGRQ scores at 12 months between the randomised groups. We will use multiple linear regression, initially with adjustments for baseline SGRQ score and country and in subsequent analysis with adjustments for other factors including age, gender and disease severity. We will estimate the difference in means with a one-sided 80% confidence interval. We will examine repeated measurements of SGRQ by treatment group and fit random effects models to consider the effect of the intervention over time. The main analysis will be by intention-to-treat (ITT), based on cases where the primary outcome is available and will, therefore, rely on an assumption that data is missing at random. We will describe the number (%) with missing primary outcome, look at reasons for missingness and consider characteristics of the patients excluded from the ITT analysis. Models will be rerun including adjustment for factors found to be related to missingness of the 12-month score. In pre-specified exploratory analysis we will examine differences by site, and by existing vs. new diagnosis of COPD.

#### Management

The core team (TS, SP, SQ, NR, WC, JH) report to a Trial Steering Committee (TSC) that includes representation from the funder, and other stakeholders. Independent members (including the chair) are drawn from our International Advisory Board. The TSC meets 6-monthly. There is an independent DSMB with one planned interim analysis examining safety data, reporting directly to the TSC. Our other team members run the Health Economics (MS, AM, MKC), Implementation Research (SP, AC, SM) and Data (JB) cores. Each site has a dedicated member of the core team to provide initial support and assistance. Data will be analysed biweekly by the Data core to assess for missingness and outliers.

#### Economic evaluation

This analysis will primarily aim to evaluate the cost-effectiveness of a multi-faceted intervention centred on a self-management strategy for COPD exacerbations within the effectiveness-implementation trial. However, in an attempt to integrate implementation science concepts with decision analysis, we will also incorporate health system factors relating to service provision in the analysis for each of the three settings and explore equity implications. The main analysis will compare the health-related costs and benefits of the COPD Action Plan plus education with the health-related costs and benefits of default standard care, as observed within the trial. Costs will be calculated using the reported levels of resource use and multiplying these estimates by the unit costs for each resource. The EQ-5D results will provide estimates of the effectiveness in terms of QALYs. QALY tariffs will be taken from the country itself, when available, or from another relevant source (e.g. adjacent country or international average). Cost-effectiveness ratios will be reported as the additional cost per QALY gained; however, these will also be reported as additional cost per hospitalisation and exacerbation averted to provide a clear picture of the value of the intervention. As COPD is expected to affect the labour status and productivity of working-age people and their caregivers, we will additionally explore the broader productivity benefits. The main cost-effectiveness analysis will further include a sensitivity analysis that accounts for the performance of the case-finding questionnaire and which extends to the short-term costs using assumptions and evidence of future costs not captured within the GECo study.

To explore equity in the cost-effectiveness analysis, we will assess whether there are population and individual characteristics that enable some sub-groups to gain more from the intervention than others. With the benefit of intense follow-up and monitoring at multiple time periods, we will be able to explore whether the intervention provides equity benefits over the course of implementation. Equity will be assessed according by examining differences in the effectiveness of the intervention according to socioeconomic sub-groups. Within the trial, different health system factors, or ‘constraints’, may hinder access, utilisation or service provision and affect the cost-effectiveness of the COPD Action Plan. Examples of constraints include: access to drugs for the management of exacerbations and access to emergency care for severe complications, which work through factors such as health insurance coverage or distance to health facilities. We will also identify constraints through the implementation science outcomes related to acceptability and feasibility. For this reason, the second part of this work will explore how constraints interact with the value of the COPD Action Plan, in the health system in each setting.

The results from these analyses will be:

1. Establish whether the intervention is cost-effective and to what degree it provides labour and productivity benefits, thus informing decisions for investment and scale-up
2. Identify important equity concerns so that any trade-offs between maximising health and maximising fairness when scaling up the intervention are made explicit, and
3. Identify the important health system constraints that future implementation efforts should consider in order to maximise COPD Action Plan value

### Implementation outcomes

#### Acceptability

We will conduct key informant in-depth interviews to evaluate acceptability of the intervention from the perspectives of local community members, CHWs, local healthcare professionals, and Ministries of Health over the course of the trial. We will also ask individuals with COPD to evaluate satisfaction with individual components of the intervention in improving quality of life and ability to manage their COPD quarterly.

#### Feasibility

We will solicit perspectives from the key groups mentioned above regarding the feasibility of the intervention during planned in-depth interviews and focus group discussions quarterly. In addition, we will record and evaluate overuse of rescue packs, which can result in antibiotic resistance and thus limit the feasibility of use in these settings. We will also ask CHWs to maintain a log of all visits, contacts from participants and lengths of these interactions throughout the follow-up period.

#### Fidelity

We will assess fidelity to the intervention during all phases of implementation of GECo2. First, we will use an LMIC-adapted version of the COPD-Q to assess knowledge of COPD on the part of CHWs and study participants to determine the effectiveness of the COPD education curriculum [37]. We will administer the questionnaire before and after the initial training of CHWs and assess change in COPD-Q score to assess score improvement. We will also administer this questionnaire to all study participants before and after their initial education session with the CHWs, as well as quarterly during follow-up visits to determine knowledge retention over time.

We will take attendance at all training sessions delivered to CHWs. CHWs will be expected to meet a set of core competencies before being allowed to deliver COPD education to study participants. Study team members will also conduct observations of the initial and follow-up visits by the CHWs. We will use checklists to determine fidelity to the intervention delivery protocols at each stage.

### Ethics

Approvals: the trial has been reviewed and approved by the University College London Research Ethics Committee (9661/001), Johns Hopkins School of Medicine (IRB00139901), Uganda National Council for Science and Technology, Makerere School of Medicine (SOMREC 2017–096), Nepal Health Research Council (136/2017) and A.B. PRIMSA (CE2147.17). Additionally, the trials have been registered at ClinicalTrials.gov (GECo1: NCT03359915, and GECo2: NCT03365713).

Community consultation: community leaders were identified and approached to obtain permission to deliver the interventions and conduct the evaluation surveys. Orientation meetings were held to explain the project aims, and community leaders will be invited to observe the randomisation process at the start of the trial.

Consent: individual participation in any part of the case-finding (GECo1) and/or self-management trial (GECo2) will be on a voluntary basis, and participants can choose to withdraw from either study at any time. Prior to all data collection the purpose of the study will be explained, an information sheet will be provided, and consent from the participant will be obtained. Respondents will be told that they can decline to participate in the study and can refuse to answer any question. Access to the identifiable individual-level data will be restricted to local study staff. All participants provide signed informed consent.

There are risks concerning self-administration of corticosteroids and antibiotics in the community setting. The risk of administration of short-term corticosteroids is minimal and patients with a history of pulmonary tuberculosis undergoing treatment will be excluded. The largest risk with antibiotics is drug reaction, though antibiotic resistance due to overuse is an additional risk. Participants who experience a drug reaction will be instructed to discontinue the medication and contact the local study investigator. Based on the severity of reaction, they will be referred to the local health centre. We will take detailed data regarding frequency and duration of all medication use during the duration of the intervention.

### Role of funder

This study is funded by the UK MRC (Medical Research Council) under a Global Alliance for Chronic Disease (GACD) call. Peer review of the original grant application contributed to the final design of the study. A representative of the funder is in attendance at the TSC. The funder otherwise has no role in the conduct or analysis of the study.

### Dissemination

The results of the study will be submitted for publication in peer-review journals, and for presentation at international meetings. We anticipate two primary manuscripts reporting GECo1 and GECo2, and papers reporting subsidiary analyses. Results will be presented locally at each of our sites. Results will be used to formulate policy documents to inform future provision of care for people living with COPD. The GECo studies are active on Twitter (@COPDGECo), and there is a trial website (https://www.globalncd.org/geco-trial) providing updates on progress.

## Discussion

This paper describes the study protocol of the GECo group: Implementation of COPD Case Finding and Self-Management Action Plans in Low and Middle Income Countries. Study investigators will engage a diverse set of stakeholders through this process including local Ministries of Health, pulmonary specialists, primary care physicians, CHWs and, importantly, individuals with COPD to ensure that the stated interventions are acceptable and appropriate to local environments. If successful this study will increase our understanding of the efficacy and implementation of case-finding and self-management strategies in diverse sites in Asia, South America and Sub-Saharan Africa with the aim of scaling this intervention in a range of LMIC settings.

### Trial status

The trial is on-going. Enrolment for GECo1 began on 5 January 2018 and GECo2 on 8 February 2018.

## Additional file

Additional file 1: SPIRIT 2013 Checklist: Recommended items to address in a clinical trial protocol and related documents*. (DOC 254 kb)

## Abbreviations

CAT: COPD Assessment Test
CHW: Community health worker
COPD: Chronic obstructive pulmonary disease
COPD-Q: COPD Questionnaire
EQ-5D: EuroQol-5D
GECo: Global Excellence in COPD Outcomes
LFQ: Lung Function Questionnaire
LMICs: Low- and middle-income countries
mMRC: Modified MRC Dyspnoea Scale
PEF: Peak expiratory flow
QALY: Quality-adjusted life year
SGRQ: St. George’s Respiratory Questionnaire
